# Comprehensive Study of Arrangement of Renal Hilar Structures and Branching Pattern of Segmental Renal Arteries: An Anatomical Study

**DOI:** 10.7759/cureus.42165

**Published:** 2023-07-19

**Authors:** Soumitra Trivedi, Urvi Sharma, Mrithunjay Rathore, Meryl R John

**Affiliations:** 1 Anatomy, All India Institute of Medical Sciences, Raipur, Raipur, IND; 2 Anatomy, Manipal Tata Medical College, Jamshedpur, IND

**Keywords:** partial nephrectomy, segmental artery, renal artery, renal hilum, kidney

## Abstract

Background

The knowledge of renal artery (RA) and its segmentation is critical before attempting any surgical procedure of kidney. The RA receives blood supply from various arteries during its descent in the embryonic period. As a result, the segmental RAs show a lot of variability in the site and pattern of origin as well as its point of entry in the kidney.

Aim/objective

The aim is to study the variable pattern existing in different segmental RAs and the arrangement of structures at the renal hilum.

Methods

The RA of 205 kidneys (68 paired and 69 unpaired) was studied and the segmental pattern was exclusively observed in cadavers by dissecting 161 kidneys, making resin/silicon casts of 34 kidneys and radiological imaging of 10 kidneys.

Results

The results obtained were quite significant and provided in detail understanding of the five main segmental arteries and the arrangement of structures at the renal hilum. Great variations were seen in the disposition of structures at the renal hilum. Six different patterns of structures at the renal hilum were obtained. Pattern 1 was the commonest pattern with an incidence of 30.3% followed by pattern 2. The site of origin of segmental arteries and their point of entry into the kidney were recorded and tabulated. The segmental arteries were classified into different types based on the frequency of their occurrence in decreasing order. In each of them, Type 1 is the commonest variant seen based on the origin of the apical artery (A), anterior upper segmental artery (AU), anterior middle segmental artery (AM), lower segmental artery (L), and posterior segmental artery (P).

Conclusions

The arrangement of hilar structures has been classified into six patterns and the variations existing in each of the segmental branches of the RA have been categorized as well. The knowledge will be invaluable for accurate radiographic interpretation of the renal vasculature and effective surgical planning in cases involving kidney transplantation, renal trauma, and partial nephrectomy. Furthermore, it will serve to prevent complications during surgical procedures.

## Introduction

Each kidney has a bean-shaped configuration with a concave medial margin and a convex lateral margin. The arrangement of structures in the hilum is renal vein (RV), renal artery (RA), and renal pelvis (P) from front to back [1]. The placement of renal hilar structures, however, varies according to reports. Around 70% of people only have one RA [2]. The posterior tributary of the renal vein (PT) and the posterior division of the RA (PD) may occasionally be observed entering the hilum posterior to the pelvis [3]. The RA and RV are clamped separately since combined clamping might induce an arterio-venous fistula [4]. As hilar dissection is a necessary step in endopyelotomy surgery, surgeons should be familiar with the arrangement of the structures at the renal hilum [5]. Therefore, prior to any urological surgical procedures, it is imperative to have a clear understanding of the normal and variant architecture of the structures at the renal hilum [6]. An understanding of the arrangement of structures at the renal hilum will provide immense help to the urosurgeons in performing different surgical procedures and prevent complications related to the surgery [1].

During the process of removing calculus from the calyx, Graves encountered two cases in 1952 where chronic hematuria ensued, prompting him to perform a complete nephrectomy. This urged him to explore the distribution of arteries in the kidney [7]. During development, kidneys ascend from the pelvis, and they are supplied by vessels that are near them. Below the origin of the superior mesenteric artery, paired RA diverges in a lateral direction from the aorta. Each RA splits into AD and PD, which then split into segmental arteries, which serve different renal vascular segments [8]. AD gives apical, upper, middle, and lower segmental arteries and supplies the majority of the kidney while the PD gives the posterior segmental artery which supplies the posterior segment of the kidney. Graves' study on the segmental pattern distribution of RA was supported by some authors [9,10]. It is crucial to keep in mind that RA branches are end arteries that only supply blood to a portion of the kidney parenchyma and do not have intersegmental anastomosis. Thus, a blockage in one of them may result in segmental ischemia and consequent hypertension. Contrarily, the veins show free anastomosis that makes it simple to knot them. Understanding the differences between the numerous segmental arteries emerging from RAs gives important clinical data, particularly for surgery and radiography [8].

Although there are a limited number of studies describing the arrangement of renal hilar structures [6], we assessed the positioning of the renal hilar structures in the central Indian population, taking into account the significance of hilar structure arrangement during surgery, particularly laparoscopic partial nephrectomy and renal transplant surgeries. It has been noticed that the arrangement of structures near the hilum frequently varies. There are several published case reports [8,11,12] stating that the structure's placement at the hilum varies. However, according to the literature, no systematic study has been done describing the normal and variable anatomy of hilar structures and segmental arteries in the central India population. So, the aim of the present study is to study the arrangement of structures at the hilum and the pattern of segmental arteries in the central India population. The current study about the variations of the segmental branches of the RA is done because of its significance in achieving a relatively bloodless surgical approach and in preserving the renal segments in partial nephrectomy [13]. Previous research on renal vascular segmentation and the number of segmental arteries has produced conflicting results [7,10,14-19]. Numerous patterns reported in the literature lead to perplexity among clinicians.

## Materials and methods

The present study was done by collecting data over a period of timeline from 2005 to March 31, 2023 by thorough examination and observing the renal hilar structures and segmental branching pattern of RA at hilar and prehilar regions in the kidneys obtained from cadavers while routine dissection in Anatomy Department. Specimens were dissected carefully after reflecting the loops of small and large intestines lying in front of the kidneys. Meticulous dissection was done to expose the retroperitoneal aorta, renal vessels, and pelvis at the hilar and prehilar regions. While dissecting the cadavers, kidneys were removed with or without the portion of the aorta keeping intact the renal hilar arrangement. The arrangement of structures at the hilum and segmental pattern was also observed by further dissection in all the renal specimens already present in the department. The total number of kidneys studied was 205 (68 paired and 69 unpaired) which were all obtained from cadavers and specimens of the Anatomy department. Further, one of the following three methods was applied for the study of branching patterns of renal segmental arteries.

Cadaveric dissection of renal segmental arteries was done in 161 kidneys. The kidneys and the structures at the hilum were dissected and observed for their arrangement in relation to standard book references. Further, the segmental arteries were tracked and dissected all the way inside the kidneys. The renal tissue surrounding the segmental arteries was removed in piecemeal to expose their path till they dichotomously divide into arcuate arteries.

Forty-four kidneys obtained from cadavers were studied by methods other than dissection to obtain clear specimens or images of segmental arteries. To prepare the cast or take a radiographic image of the RA and its segmentation the vessel has to be cleaned prior to injecting resin, silicon, or the dye. The passage was cleared by passing tap water through the RA for two hours, followed by two liters of 0.9% NaCl solution. After this, three to four drops of 1% HCL was added to two liters of 0.9% NaCl solution and was passed through RAs to remove any stubborn clots.

Resin and silicon cast were prepared for 34 kidneys. The RAs were injected with 5 to 10 mL of either plastic resin solution or silicon gel. It was left overnight to set and harden inside the arteries. After this, the kidneys were completely submerged in 30% HCL for one to three days, which dissolved most of the renal tissue leaving behind the arterial cast. A few of the renal tissue tags on the segmental arteries were removed under slow-running tap water. RA radiograph was taken in about 10 kidneys immediately after injecting Trazograph 76% dyes in the RA of the specimens.

## Results

**Figure 1:**
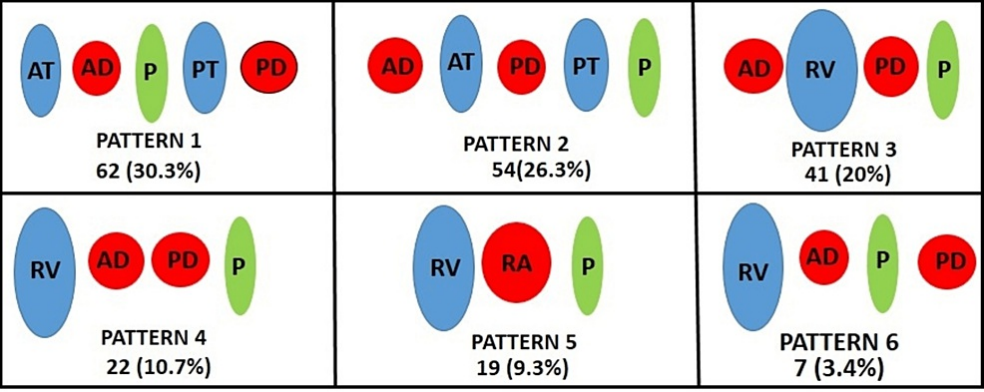
Schematic diagram showing six patterns of Hilar arrangement of structures observed anterior to posterior while entering/leaving the kidney. RA - Renal artery, RV - Renal vein, P - Pelvis, AT - Anterior Tributary of Renal vein, PT - Posterior Tributary of Renal vein, AD - Anterior Division of Renal artery, PD - Posterior Division of Renal artery

The origin of the RA, its arrangement at the hilum with other structures namely the renal vein and pelvis is quite variable (Figure 1). This is due to the fact that the kidney descends from the abdomen to the pelvis and during this descent, several arteries appear and disappear to supply kidneys. While doing studies and collecting data from 205 kidneys (68 paired and 69 unpaired), we noticed the variability in the structures at the hilum, the branching pattern of RA, and variations in the segmental RAs. A total of 97 kidneys were from the right side and 108 kidneys from the left side. Based on the arrangement of structures anterior to posterior at the hilum six groups were classified in decreasing order of frequency (Table 1, Figure 1).Pattern 1 - Anterior tributaries of renal vein - Anterior division of RA - pelvis - posterior tributary of renal vein - posterior division of RA. Pattern 2 - Anterior division of RA - Anterior tributaries of renal vein - posterior division of RA - posterior tributary of renal vein- pelvis. Pattern 3 - Anterior division of RA - Renal Vein - posterior division of RA - pelvis. Pattern 4 - Renal vein - Anterior division of RA - posterior division of RA - pelvis. Pattern 5 - Renal vein - RA - pelvis. Pattern 6 - Renal vein - Anterior division of RA - pelvis - posterior division of RA.

**Table 1:** Different patterns observed at renal hilum.

Patterns		Number of kidneys N=205	Incidence (%)
Pattern 1	Right	16	16.5
	Left	46	42.6
	Total	62	30.3
Pattern 2	Right	30	30.9
	Left	24	22.2
	Total	54	26.3
Pattern 3	Right	21	21.6
	Left	20	18.5
	Total	41	20
Pattern 4	Right	15	15.4
	Left	7	6.5
	Total	22	10.7
Pattern 5	Right	10	10.3
	Left	9	8.3
	Total	19	9.3
Pattern 6	Right	5	5.1
	Left	2	1.9
	Total	7	3.4

**Figure 2:**
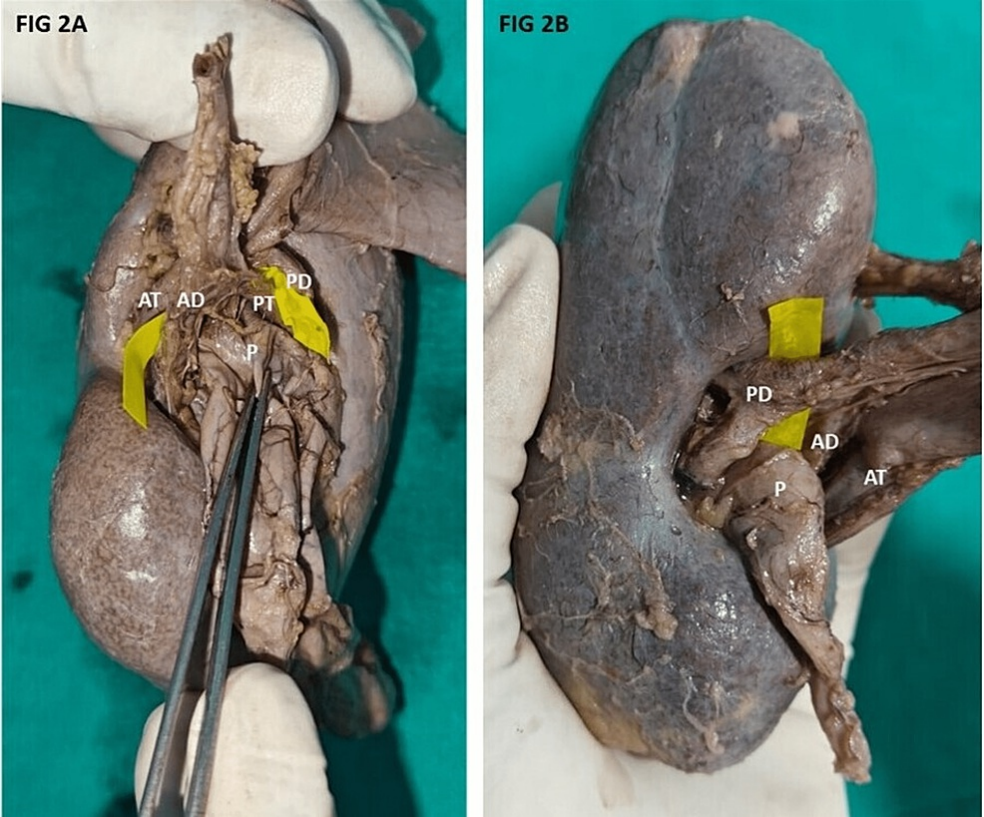
(A) Most common pattern of hilar arrangement seen anterior to posterior in the right kidney. (B) Most common pattern of hilar arrangement seen posterior to anterior in the left kidney. AT - Anterior tributary of renal vein, PT - Posterior tributary of renal vein, AD - Anterior division of renal artery, PD - Posterior division of renal artery

**Figure 3:**
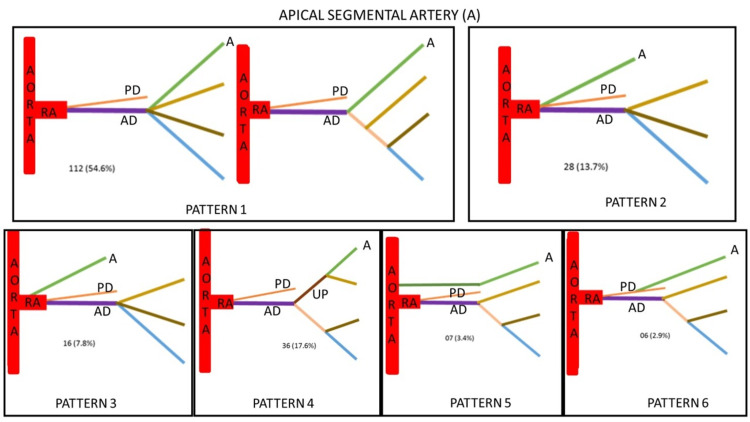
Apical segmental artery (A) showing six variant patterns of its origin. Type 1 - Arising as segmental branch directly from anterior division (AD) Type 2 - Arising as segmental branch from the junction of anterior and posterior division (PD) Type 3 - Arising as segmental branch directly from renal artery (RA) Type 4 - Arising from the upper presegmental branch (UP) of anterior division Type 5 - Arising as segmental branch directly from aorta Type 6 - Arising as segmental branch from the posterior division (PD) of renal artery

Pattern 2, pattern 3, pattern 5 and pattern 6 type of arrangement was observed on the right side while pattern 1 was observed on the left side. Figures 2A, 2B showing the most common type of hilar arrangement. Further in this study, the five segmental arteries and their variant patterns were observed based on the site of origin and the variant pattern was studied with different methods. Apart from dissection, radiographs and resin cast were prepared to look for the variation in the origin and branching patterns of the segmental arteries. Based on the origin of the apical artery and its variations, it was classified into six types (Figure 3).

**Figure 4:**
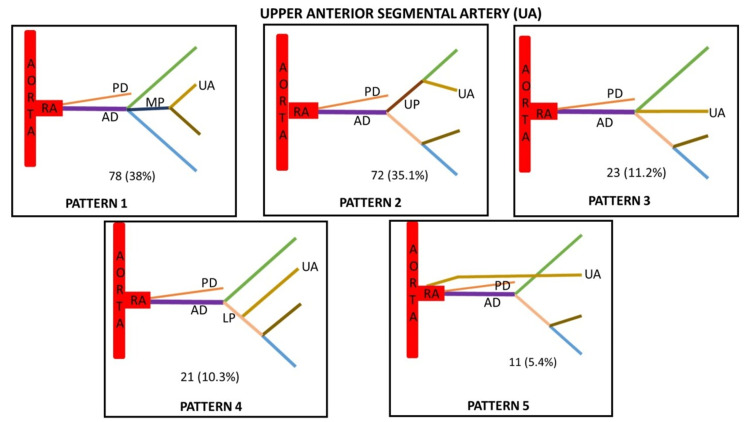
Upper anterior segmental artery (UA) showing five variant patterns of its origin. Type 1 - Arising from the middle presegmental branch (MP) of anterior division Type 2 - Arising from the upper presegmental branch (UP) of anterior division Type 3 - Arising as segmental branch directly from anterior division (AD) Type 4 - Arising from lower presegmental branch (LP) of anterior division Type 5 - Arising as segmental branch directly from renal artery (RA)

Based on the origin of anterior upper segmental artery and its variations, it was classified into five types (Figure 4).

**Figure 5:**
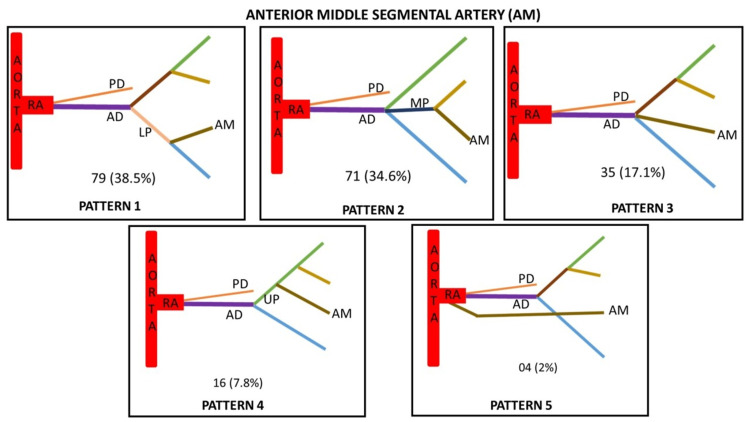
Anterior middle segmental artery (AM) showing five variant patterns of its origin. Type 1 - Arising from lower presegmental branch (LP) of anterior division Type 2 - Arising from the middle presegmental branch (MP) of anterior division Type 3 - Arising as segmental branch directly from anterior division (AD) Type 4 - Arising from upper presegmental branch (UP) of anterior division Type 5 - Arising as segmental branch directly from renal artery (RA)

Based on the origin of anterior middle segmental artery and its variations, it was classified into five types (Figure 5).

**Figure 6:**
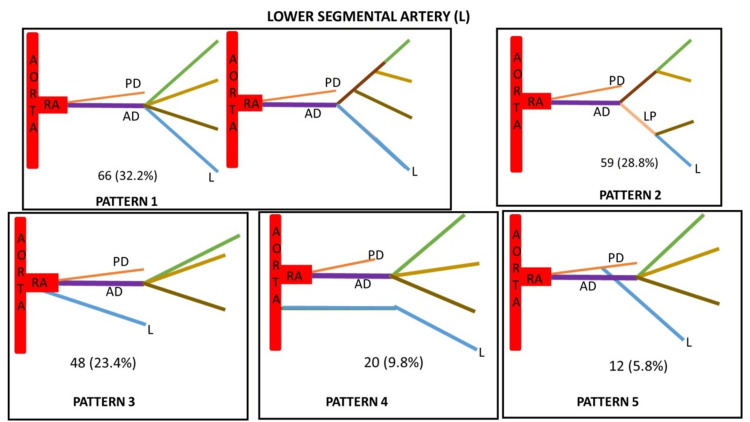
Lower segmental artery (L) showing five variant patterns of its origin. Type 1 - Arising directly from the anterior division (AD) as segmental artery Type 2 - Arising from the lower presegmental branch (LP) of anterior division Type 3 - Arising directly as segmental branch from renal artery (RA) Type 4 - Arising directly as segmental branch from aorta Type 5 - Arising directly as segmental branch from the posterior division (PD)

Based on the origin of lower segmental artery and its variations, it was classified into five types (Figure 6).

**Figure 7:**
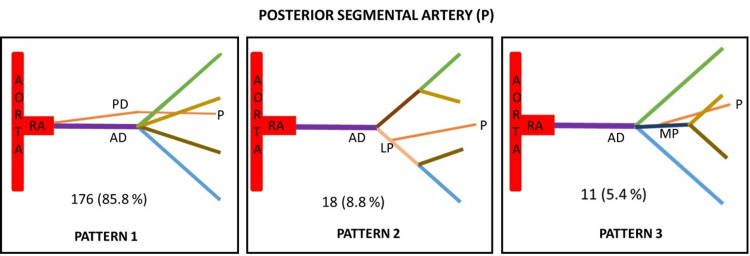
Posterior segmental artery (P) showing three variant patterns of its origin. Type 1 - Arising from the posterior division (PD) as its continuation Type 2 - Arising from lower presegmental branch (LP) of renal artery Type 3 - Arising from the middle presegmental branch (MP) of renal artery

Based on the origin of posterior segmental artery and its variations, it was classified into three types (Figure 7).

**Figure 8:**
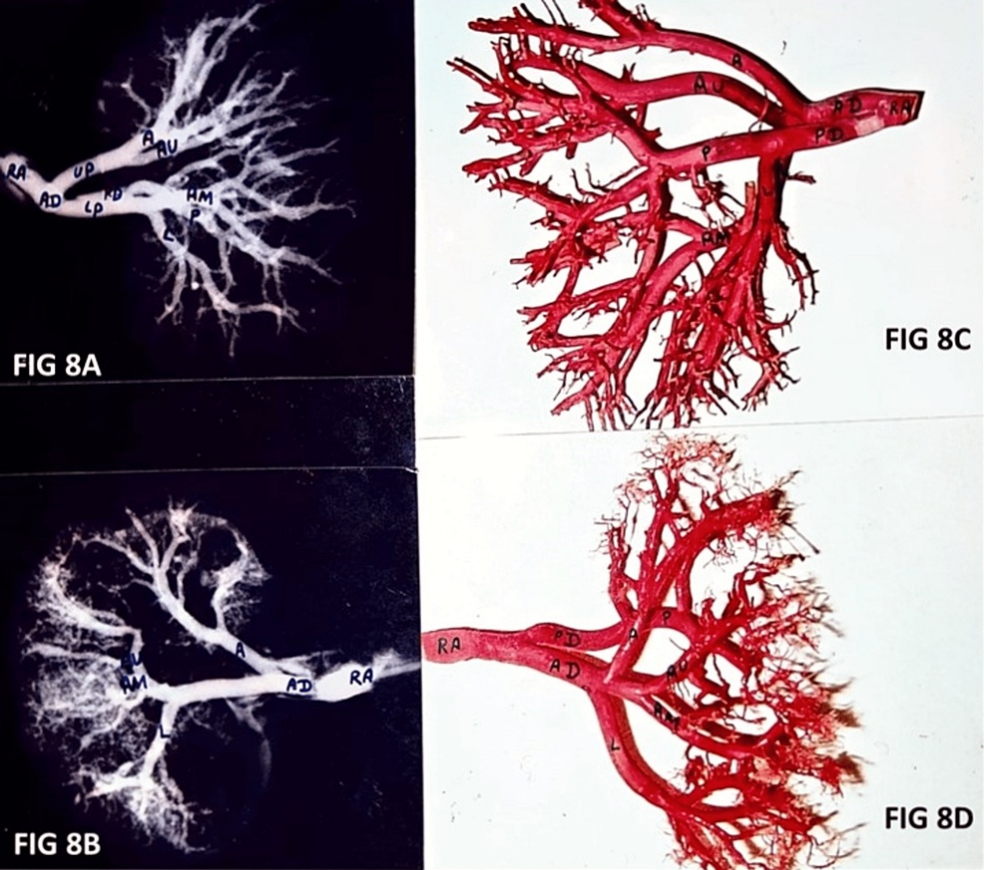
Radiographic images and resin casts showing pattern of different segmental arteries. RA - Renal artery, AD - Anterior division of renal artery, PD - Posterior division of renal artery (A) Radiographic image showing segmental pattern of left renal artery seen in AP view. (B) Radiographic image showing segmental pattern of right renal artery seen in AP view. (C) Resin cast image showing segmental pattern of left renal artery seen from posterior aspect. (D) Resin cast image showing segmental pattern of left renal artery seen from anterior aspect.

Different segmental arterial patterns were observed while making resin casts and taking radiographic images of renal arteries (Figure 8). The segmental pattern of various branches was decided by their site of origin. One of the radiographic images of segmental pattern of left Renal artery showing Type 4 pattern of Apical, Type 2 pattern of anterior upper and lower segmental artery and Type 1 pattern of anterior middle and posterior segmental artery (Figure 8A) whereas another radiographic image of segmental pattern of right renal artery showing Type 3 pattern of apical, Type 1 pattern of anterior upper, lower and posterior segmental artery and Type 2 of anterior middle segmental artery (Figure 8B). Resin casts were also made and the images were taken to observe the segmental patterns one of which shows a left renal artery having Type 1 pattern of apical, Type 3 of anterior upper and anterior middle, Type 5 of lower and Type 1 of posterior segmental artery (Figure 8C). Another resin cast image of segmental pattern of left renal artery showing Type 1 pattern of apical, anterior upper, lower and posterior segmental artery and Type 2 pattern of anterior middle segmental artery (Figure 8D).

Based on the site of origin, the fives segmental arteries were classified into types in decreasing order of their frequency (Table 2). The maximum variations were seen in apical segmental artery followed by anterior upper, anterior middle and lower segmental arteries. The posterior segmental arteries were least variable.

**Table 2:** Pattern and incidence of different segmental arteries.

Types	Branching patterns				
	Apical artery N=205	Anterior Upper segmental artery N=205	Anterior Middle segmental artery N=205	Lower segmental artery N=205	Posterior segmental artery N=205
Type 1 (%)	112 (54.6)	78 (38)	79 (38.5)	66 (32.2)	176 (85.8)
Type 2 (%)	36 (17.6)	72 (35.1)	71 (34.6)	59 (28.8)	18 (8.8)
Type 3 (%)	28 (13.7)	23 (11.2)	35 (17.1)	48 (23.4)	11 (5.4)
Type 4 (%)	16 (7.8)	21 (10.3)	16 (7.8)	20 (9.8)	-
Type 5 (%)	7 (3.4)	11 (5.4)	4 (2)	12 (5.8)	-
Type 6 (%)	6 (2.9)	-	-	-	-

## Discussion

The renal hilum is a vertical slit on the kidney's medial border. In the hilum, structures arranged anteroposteriorly are vein-artery-pelvis [2]. There are very few studies describing the arrangement of structures at the hilum of the kidney [6]. The present study found six distinct types of structural arrangements at the renal hilum and the type 1 pattern (30.3%) was more prevalent followed by type 2 (26%), type 3 (20%), type 4 (13%), type 5 (9%) and type 6 (3%) (Table 3). A total of 10 distinct patterns of hilar structural arrangements in the anteroposterior direction were noted in this study, whereas some other researchers noted 12 different types of patterns at the renal hilum [6,20]. In our study, in pattern 5, the normal arrangement of the hilar structures is noted while the remaining pattern shows variation in the arrangement of hilar structures. We have compared the results of the present study with the earlier studies, and we observed that authors got variable patterns and their incidence. It is because renal vessel variations are common in the population with discrete prevalence among racial and ethnic groups [6]. In the present study variant pattern is commonly seen on the right side similar to Jadhav and Zambare. In contrast, in one study, the variant pattern has been noted on the left side (Table 4) [20]. It has been observed that in most cases, branches and tributaries of renal vessels occupy the hilar and prehilar area rather than the main division of RAs, and variation in their branching pattern is primarily responsible for such arrangements [6]. The defect in the rotation of the kidney may also result in a variant arrangement of hilar structures which leads to ureteropelvic obstruction [21]. In 29%-65% cases, ureteropelvic obstruction is caused due to variation in the arrangement of renal vessels [21]. Obstruction at the ureteropelvic junction leads to upper urinary tract obstruction [20]. In 66.3%, the kidney's pelvis was the posterior-most structure. However, a study done in the Bangladesh population noted renal pelvis is the posterior-most structure in 100% of the kidneys [22]. Before performing various procedures, urologists need to have a thorough understanding of the arrangement of the renal hilar structures, as they will be performing hilar dissection and hilar vascular clamping [10].

**Table 3:** Arrangement of renal structures at hilum in various studies AT - Anterior tributary of renal vein, PT - Posterior tributary of renal vein, AD - Anterior division of renal artery, PD - Posterior division of renal artery, RA - renal artery, RV - renal vein, P - Pelvis

Pattern	Arrangement of structures at renal hilum					
	Patterns	Trivedi et al.-2011 (%) [3]	Kumar et al.-2013 (%) [20]	Jadhav and Zambare-2015 (%) [6]	Varalakshmi and Sangeeta-2017 (%) [1]	Present study (%)
1	AT-AD-P-PT-PD	22	-	-	-	30.3
2	AD-AT-PD-PT-P	-	-	-	-	26.3
3	AD-RV-PD-P	23	2.1	5.26	17.18	20
4	RV-AD-PD-P	8	-	-	-	10.7
5	RV-RA-P	19	45.8	22.80	31.24	9.3
6	RV-AD-P-PD	20	8.3	7.89	21.87	3.4

**Table 4:** Arrangement of structures at renal hilum on right and left kidney in various studies

Studies	Pattern 1		Pattern 2		Pattern 3		Pattern 4		Pattern 5		Pattern 6	
	Right (%)	Left (%)	Right (%)	Left (%)	Right (%)	Left (%)	Right (%)	Left (%)	Right (%)	Left (%)	Right (%)	Left (%)
Kumar et al. (2013) [20]	-	-	-	-	2.3	1.8	-	-	47.61	44.44	7.14	9.25
Jadhav and Zambhare (2015) [6]	5.26	5.26	-	-	5.26	5.26	-	-	21.05	24.56	10.5	5.26
Present study	16.5	42.6	30.9	22.2	21.6	18.5	15.4	6.5	10.3	8.3	5.1	1.9

During nephrectomy, information about the structures arranged at the hilum of the kidney is essential for urosurgeons. In such cases, clamping of distinct hilar structures is recommended over bulk clamping as it may lead to complications like arteriovenous fistula [4]. Information on the arrangement of hilar structures is also essential in procedures like laparoscopic pyelolithotomy [23]. A discrete pattern of structures at the renal hilum may perplex surgeons and may cause difficulty while performing surgery at the hilum region which may lead to iatrogenic injury which can cause emergency during laparoscopic partial nephrectomy. Thus, having a thorough understanding of renal hilar structures is crucial. Laparoscopic surgery is more difficult than open surgery during hilar dissection [24,25]. Due to variations in the arrangement of hilar structures, the earlier studies have prioritized lateral deep incisions rather than posterior or anterior incisions along the ureteropelvic junction in endopyelotomies [26].

A precise anatomical understanding of renal segmentation is required due to the development of an increasing number of conservative procedures in the field of endourological surgery [27]. We have studied different segmental arteries based on the origin of the apical artery, anterior upper segmental artery, anterior middle segmental artery, lower segmental artery, and posterior segmental artery. The present study identified six different patterns of Segmental arteries based on the origin of apical arteries (Table 5). The incidence of type 1 is 54.6% which is similar to the study conducted by Raghavendra et al. [27] in which the incidence is 51.6%. In contrast, a study was conducted where the incidence reported was 20.4% [28]. The incidence of type 2 in the present study is 17.6% which is similar to the study by yet another researcher which was 15% [29]. The incidence of type 6 is slightly higher in a study conducted by Kher et al. as compared to other studies [16,28,29]. The incidence of type 5 in the present study is 3.4% which is similar to the study conducted by Kher et al. [28], i.e., 2.45%. The incidence of type 6 in our study is less as compared to other studies. Based on the origin of the lower segmental artery, the incidence of type 1 is 32.2%, which is slightly less as compared to other studies while the incidence of type 3 is higher in our study (Table 6). The incidence of type 4 is higher in a study conducted by Fine and Keen [10]. Based on the origin of the posterior segmental artery, the incidence of type 1 in our study is 85.8%, which is similar to the study conducted by Kaushik et al. [8] while the incidence of type 2 and type 3 in the present study is 18% and 11%, respectively. Double posterior segmental artery was observed in 2.14% cases in a study conducted by Fine and Keen [10]. Thampi and Krishnapillai noticed an absent posterior segmental artery in six out of 48 kidneys [30]. No study describing the anterior upper segmental and anterior middle segmental artery was found in the literature.

**Table 5:** Comparative study on the apical segmental artery

Authors	Kher et al.-1960 (%) [28]	Verma et al.-1961 (%) [29]	Raghavendra-2007 (%) [27]	Thampi et al.- 2017 (%) [30]	Present study
Sample size	54	98	60		205
Type 1	45.2	20.4	51.6	43.47	54.6
Type 2	15	-	25	-	17.6
Type 3	5.6	16.3	1.6	13.04	13.7
Type 4	1.86	29.7	11.6	10.14	7.8
Type 5	2.45	-	1.6	-	3.4
Type 6	29.7	-	8.3	21.73	2.9

**Table 6:** Comparative study of lower segmental artery

Authors	Kher et al. -1960 (%) [28]	Verma et al.-1961 (%) [29]	H.Fine-1966 (%) [10]	Present study-2023 (%)
Sample size	54	98	107	205
Type 1	74	88	-	32.2
Type 2	-	-	-	28.8
Type 3	1.85	1.02	-	23.4
Type 4	1.85	3.06	38	9.8
Type 5	20	1.02	-	5.8

After the depiction of Brodel's line in 1901, renal operations have undergone a revolution. Since then, many sophisticated renal reconstructive techniques have been developed to preserve the renal parenchyma as a substitute for radical nephrectomy. The modern procedures used in renal operations depend on segmental resection. Wedge-type resections are performed when either the upper or lower segments of the kidney are affected. However, if the middle segment is involved, a partial nephrectomy is carried out. The absence of arterial anastomosis in the adjacent segments will only affect the damaged segment, without disrupting blood flow to the surrounding segments or causing ischemia. Since there is no arterial anastomosis, the resection procedure will be simpler as the region of the surgery will be mostly bloodless once the segmental artery supplying it has been severed. The ideal direction to do segmental resection is from the hilum to the periphery. The upper segmental branch may occasionally need to be sacrificed in type 2 situations by the surgeons. The type 3 cases provide greater challenges for the surgeons when ligating this segmental branch and resecting the artery since ligating may affect the adjacent segments. The inferior suprarenal artery may emerge from the superior accessory RA in type 5 situations. The segmental arteries that supply the apical area typically emerge from the posterior division in type 6 instances, which makes it simpler for the surgeons to perform ligations of those arteries (16).

## Conclusions

The present study has provided the six different types of patterns of arrangement of structures at the renal hilum. It also documented variations in the origin of the segmental arteries like apical, anterior upper segmental, anterior middle segmental, lower, and posterior segmental arteries. Pattern 2 of hilar arrangement is observed in our study which was not described previously in any other study. As described in the standard books, the typical pattern of hilar arrangement was observed in 9.3% of the population (pattern 5). The variant pattern in the origin of five segmental arteries was described in which the apical segmental artery was classified into six types, anterior upper, anterior middle, and lower were classified into five types each, and posterior was classified into three types. Out of these variant patterns, the origin of the anterior upper and anterior middle has been described for the first time in detail in this study, and there is no literature available regarding these segmental branches. Due to high variability in the arrangement of structures at the renal hilum, knowledge of the variant anatomy will be helpful in renal transplants, and laparoscopic surgery to avoid complications during surgery. Incidence of pattern in segmental arteries shall aid in the planning and execution of surgical interventions in situations of renal transplant, selective clamping in cases of partial nephrectomy, and selective embolization of arteries in renal vascular injuries after operations.

## References

[1] Varalakshmi KL, Sangeeta M (2017). A cadaveric study on dimensions and hilar structural arrangement of kidney. Int J Anat Res.

[2] Standring S (2005). Gray’s Anatomy, 39th Edition: The Anatomical Basis of Clinical Practice. AJNR Am J Neuroradiol.

[3] Trivedi S, Athavale S, Kotgiriwar S (2011). Normal and variant anatomy of renal hilar structures and its clinical significance. Int J Morphol.

[4] Rapp DE, Orvieto MA, Gerber GS, Johnston WK 3rd, Wolf JS Jr, Shalhav AL (2004). En bloc stapling of renal hilum during laparoscopic nephrectomy and nephroureterectomy. Urology.

[5] Sampaio FJ, Favorito LA (1991). Endopyelotomy. Anatomical study of the vascular relationships to ureteropelvic junction (Article in French). J Urol (Paris).

[6] Jadhav S, Zambare B (2015). Anatomical study of arrangement of renal hilar structures in Indian adult human cadavers. Natl J Integr Res Med.

[7] Graves FT (1954). The anatomy of the intrarenal arteries and its application to segmental resection of the kidney. Br J Surg.

[8] Kaushik RK, Khare S, Jain S, Tripathi A, Kausar H, Raizaday S (2020). Study of renal posterior segmental artery by corrosion cast method. J Evid Based Med Healthc.

[9] Sykes D (1963). The arterial supply of the human kidney with special reference to accessory renal arteries. Br J Surg.

[10] Fine H, Keen EN (1966). The arteries of the human kidney. J Anat.

[11] Gesase AP (2007). Rare origin of supernumerary renal vessels supplying the lower pole of the left kidney. Ann Anat.

[12] Hazırolan T, Öz M, Türkbey B, Karaosmanoğlu AD, Oğuz BS, Canyiğit M (2011). CT angiography of the renal arteries and veins: normal anatomy and variants. Diagn Interv Radiol.

[13] Chandragirish S, Nanjaiah CM, Suhas YS, Saheb SH (2014). Study on variations of Inferior Segmental Branch of renal artery. Int J Anat Res.

[14] Weld KJ, Bhayani SB, Belani J, Ames CD, Hruby G, Landman J (2005). Extrarenal vascular anatomy of kidney: assessment of variations and their relevance to partial nephrectomy. Urology.

[15] Ajmani ML, Ajmani K (1983). To study the intrarenal vascular segments of human kidney by corrosion cast technique. Anat Anz.

[16] Sampaio FJ, Favorito LA (1993). Ureteropelvic junction stenosis: vascular anatomical background for endopyelotomy. J Urol.

[17] Sapte E, Bordei P (2005). Anatomical considerations on the renal arterial segmentation (Article in Romanian). Rev Med Chir Soc Med Nat Iasi.

[18] Shoja MM, Tubbs RS, Shakeri A, Loukas M, Ardalan MR, Khosroshahi HT, Oakes WJ (2008). Peri-hilar branching patterns and morphologies of the renal artery: a review and anatomical study. Surg Radiol Anat.

[19] Ogeng'o JA, Masaki CO, Sinkeet SR, Muthoka JM, Murunga AK (2010). Variant anatomy of renal arteries in a Kenyan population. Ann Transplant.

[20] Kumar N, Guru A, Aithal P. A, Shetty SD, Nayak B. S, Pamidi N (2013). Evaluation of the variant anatomical disposition of the renal hilar structures in South Indian adult human cadavers and its clinical implications. J Clin Diagn Res.

[21] Rouvière O, Lyonnet D, Berger P, Pangaud C, Gelet A, Martin X (1999). Ureteropelvic junction obstruction: use of helical CT for preoperative assessment--comparison with intraarterial angiography. Radiology.

[22] Hassan SM, Khalil M, Khalil M (2012). Variation in arrangement of structures at hilum of human kidney. Bangladesh J Anat.

[23] Pereira CJA, Valentim LS, Castro KF, Gasque GP, Rosario CAF, Prinz RAD (2009). Analysis of renal hilum extraparenchymal structures in Brazilian adult human cadavers. Eur J Anat.

[24] Gill IS, Colombo JR Jr, Frank I, Moinzadeh A, Kaouk J, Desai M (2005). Laparoscopic partial nephrectomy for hilar tumors. J Urol.

[25] Lattouf JB, Beri A, D'Ambros OF, Grüll M, Leeb K, Janetschek G (2008). Laparoscopic partial nephrectomy for hilar tumors: technique and results. Eur Urol.

[26] Das S, Paul S (2006). Variation of renal hilar structures: a cadaveric case. Eur J Anat.

[27] Raghavendra V, Manjappa P, Telkar A (2012). Renal apical segmental artery variations and its surgical importance. J Clin and Diag Res.

[28] Kher GA, Bhargava I, Makhni FS (1960). Intrarenal branching of the renal artery. Ind J Surg. 1960;22:563-79..

[29] Verma M, Chaturvedi RP, Pathak RK (1961). Anatomy of renal vascular segments. J Anat Soc.

[30] Thampi S, Krishnapillai R (2017). A cadaveric study of variations in origin and branching of renal arteries. J Evolution Med Dent Sci.

